# Assessment of the prognostic value of the 8^th^ AJCC staging system for patients with clinically staged prostate cancer; A time to sub-classify stage IV?

**DOI:** 10.1371/journal.pone.0188450

**Published:** 2017-11-28

**Authors:** Omar Abdel-Rahman

**Affiliations:** Clinical Oncology Department, Faculty of Medicine, Ain Shams University, Cairo, Egypt; Taipei Medical University, TAIWAN

## Abstract

**Background:**

The American Joint Committee on Cancer (AJCC) staging system (8^th^ edition) for prostate cancer has been published. The current study seeks to validate the prognostic performance of the changes in the new system among clinically staged prostate cancer patients registered within the surveillance, epidemiology and end results (SEER) database.

**Methods:**

SEER database (2004–2014) has been accessed through SEER*Stat program and AJCC 7^th^ and 8^th^ edition stages were calculated utilizing T, N and M stages as well as baseline prostatic specific antigen (PSA) and grade group. Cancer-specific and overall survival analyses according to 6^th^, 7^th^ and 8^th^ editions were conducted through Kaplan-Meier analysis. Moreover, multivariate analysis was conducted through a Cox proportional hazard model.

**Results:**

A total of 110499 patients with prostate cancer were identified in the period from 2004-2014.For cancer- specific survival according to 8^th^ AJCC, all pair wise P values for comparison were significant (<0.01) except for stage IIA vs. IIB; while for overall survival according to 8^th^ AJCC, all pair wise P values for comparison were significant (<0.02) except for stage IIIA vs. IIIB. Results of c-index assessment for cancer-specific survival for the three AJCC editions were as follows: c-index for AJCC 6^th^ edition was 0.816; c-index for AJCC 7^th^ edition was 0.897; c-index for AJCC 8^th^ edition was 0.907. For stage IVB prostate cancer (i.e.M1 disease), further sub-staging was proposed according to M1 sub-stage (i.e. M1a, M1b and M1c). Pair wise comparison between these proposed sub-stages was conducted for both cancer-specific and overall survival. For both cancer-specific and overall survival, all pair wise P values for comparisons were <0.0001.

**Conclusion:**

Compared to older staging systems (6^th^ and 7^th^), the 8^th^ system is more discriminatory. Further sub-classification of stage IV disease is suggested.

## 1. Introduction

Prostate cancer is the 2^nd^ most cancer among men and the 4^th^ most common cancer in both sexes.Approximately 1.1 million patients were diagnosed with prostate cancer in 2012 according to globocan[1]. Moreover, prostate cancer is the 5^th^ leading cause of mortality from cancer in men [2].

Principles of therapy of patients with prostate cancer have incorporated multiple domains; namely: patient domain (fitness, and co-morbidity) and tumor domain (stage and grade) [3]. Successive editions from the most common staging system for prostate cancer (the American Joint Committee on Cancer (AJCC)) system have been published reflecting progress in our understanding of prostate cancer biology and prognosis. The most recent edition (8^th^ edition) has been published in December 2016 and its implementation was delayed till January 2018 [4, 5]. Notable changes in the 8^th^ edition include: (1) pathologically organ-confined disease should be considered as pT2 and should not be classified based on extent or laterality. (2) Histologic grade should be expressed by grade grouping system. (3) T4N0M0 disease is stage IIIB rather than stage IV. (4) Introduction of newer sub-stages within the realm of stage II and stage III. Summary of the AJCC 8^th^ stage grouping for prostate cancer with comparison to previous staging systems is provided in Table 1 [6][7]. It has to be noted that the first publication proposing the grade group approach to prostate cancer was from Hopkins group [8].

**Table 1:** AJCC stage groupings 6^th^, 7^th^ and 8^th^ edition*.

Stage	AJCC 6^th^	AJCC 7^th^	AJCC 8^th^
I	T1a N0 G1	T1a-c N0 M0, PSA < 10, Gleason ≤ 6T2a N0 M0, PSA < 10, Gleason ≤ 6 T1-2a N0 M0, PSA X, Gleason X	cT1a-T2a N0M0,PSA<10, grade group 1
II	T1a G2-4, T1b-1c, T2	IIA: T1a-c N0 M0, PSA < 20, Gleason 7 T1a-c N0 M0, PSA ≥ 10 < 20, Gleason ≤ 6 T2a-b N0 M0, PSA < 20, Gleason ≤7 T2a N0 M0, PSA ≥ 10 < 20, Gleason ≤ 6 T2b N0 M0, PSA X, Gleason X IIB: T2c N0 M0, Any PSA, Any Gleason T1-2 N0 M0, PSA ≥ 20, Any Gleason T1-2 N0 M0, Any PSA, Gleason ≥ 8	IIA: cT1a-T2a, N0,M0,PSA 10–20, grade group 1 IIA: cT2b-c, N0,M0, PSA<20, grade group 1 IIB: T1-2,N0,M0,PSA<20, grade group 2 IIC: T1-2,N0,M0,PSA<20,grade group 3,4
III	T3 N0	T3a-b N0 M0, Any PSA, Any Gleason	IIIA: T1-2,N0,M0,PSA ≥20, grade group 1–4 IIIB: T3-4, N0, M0, Any PSA, grade 1–4 IIIC: Any T, N0,M0, Any PSA, grade group 5
IV	T4 or N1 or M1	T4 N0 M0, Any PSA, Any Gleason N1, Any PSA, Any Gleason M1, Any PSA, Any Gleason	IVA: Any T, N1, M0, Any PSA, Any grade group IVB: Any T, N0, M1, Any PSA, Any grade group

*PSA laboratory examination unit was ng/ml.

External validation of the prognostic significance of these changes among different population-based databases and comparing new vs. older AJCC editions would confirm its prognostic impact; moreover, it may point out potential gaps whereby further improvements in the staging system are needed. Surveillance, epidemiology and end results (SEER) database is a valid choice for this external validation as well as for exploration of future refinements given its broad coverage and rigorous quality assurance [9].

Given the presence of two versions of AJCC staging system for prostate cancer based on the method of staging (i.e. clinical versus pathological staging), the current analysis is restricted to clinically staged patients in order to ensure homogeneity of the patient population.

## 2. Objective

The objective is to validate the prognostic value of the changes put forward in the 8^th^ edition of the AJCC staging system in a cohort of patients with clinically staged prostate cancer registered within the SEER database.

## 3. Methodology

The records of this study were extracted from the SEER-18 registry [10]; in order to accomplish this, SEER*Stat software Version 8.3.4 was employed.

### a. Selection of the study cohort

The SEER database search was limited to the period from 2004–2014 (because reliable PSA data were not available before that date).To identify eligible records, the ICD-O-3/WHO 2008 category of “prostate” was selected. In order to restrict the inclusion to clinically staged patients, cases with any form of radical surgical treatment to the prostate were excluded. Cases with incomplete information about survival, TNM 6^th^ stage, PSA or Gleason grade/grade group were excluded.

### b. Data collection

Data extracted for each record included age at diagnosis, race, T, N and M stages (according to the 6^th^ edition), 6^th^ edition stage group, PSA, Gleason grade, site of distant metastatic disease (if applicable), cause-specific death classification and survival months.7^th^ and 8^th^ edition stage groups were then reconstructed for each patient according to PSA, Gleason grade/grade group and 6^th^ edition T/ N/ M stages. Through tumor extension information from the collaborative staging section, disease with microscopic bladder neck invasion (categorized as T4 in the 6^th^ edition) was down-staged to T3a (as in 7^th^ and 8^th^ editions). Grade groups were calculated based on the available Gleason scores as follows: group 1: Gleason score ≤6; group 2: Gleason score 3+4; group 3: Gleason score 4+3; group 4: Gleason score 8; group 5: Gleason score 9–10 [11].

In the current study, cancer-specific survival was defined as time from diagnosis to death from prostate cancer. Available SEER data about systemic as well as radiation therapy were limited by incompleteness and uncertainty (data were available as yes or no/unknown); thus, they were not included into the current analysis. Data about performance status of the patients as well as co-morbidity were not recorded in the SEER database.

### c. Statistical considerations

In this analysis, Kaplan-Meier analysis as well as log-rank testing was used for survival comparisons (both cancer-specific and overall survival) according to 6^th^, 7^th^ and 8^th^ editions of the AJCC. Regarding cancer-specific survival, cases that were alive at the end of the study as well as cases who died of causes other than cancer were censored. Median follow up for the whole cohort is 26 months.Post hoc Bonferroni correction for multiple survival comparisons was not implemented to avoid the risk of exaggerating a type II error (in the presence of numerous pair wise comparisons).Cox proportional hazard model was utilized to conduct multivariate analyses; and hazard ratios (with corresponding 95% CI) were produced for factors affecting cancer-specific and overall survival for the subgroup of patients with clinically localized disease as well as the subgroup of patients with advanced disease.Verification of the proportional hazard assumption was made though graphical assessment of the hazard plots. A result would be considered statistically significant if a two-tailed P value was < 0.05. Moreover, a concordance index (c-index) using both death from prostate cancer and death from any cause as the dependent variables was evaluated for each of the three AJCC editions (6^th^, 7^th^ and 8^th^). C-index was calculated through binary logistic regression followed by area under the receiver operating characteristics curve were calculated. Its value gives additional insight into the discriminatory ability of each of the AJCC staging system editions. C-index was calculated for both death from prostate cancer and death from any cause in order to give extra insight about cancer-specific survival as well as overall survival. An additional modification to the sub-staging of clinically localized disease (stage I, II, III) as well as advanced disease (stage IV) was also proposed and evaluated in this study. All of the analyses were performed through SPSS Statistics 20.0 (IBM, NY).

## 4. Results

### a. Patients, characteristics

**Fig 1:**
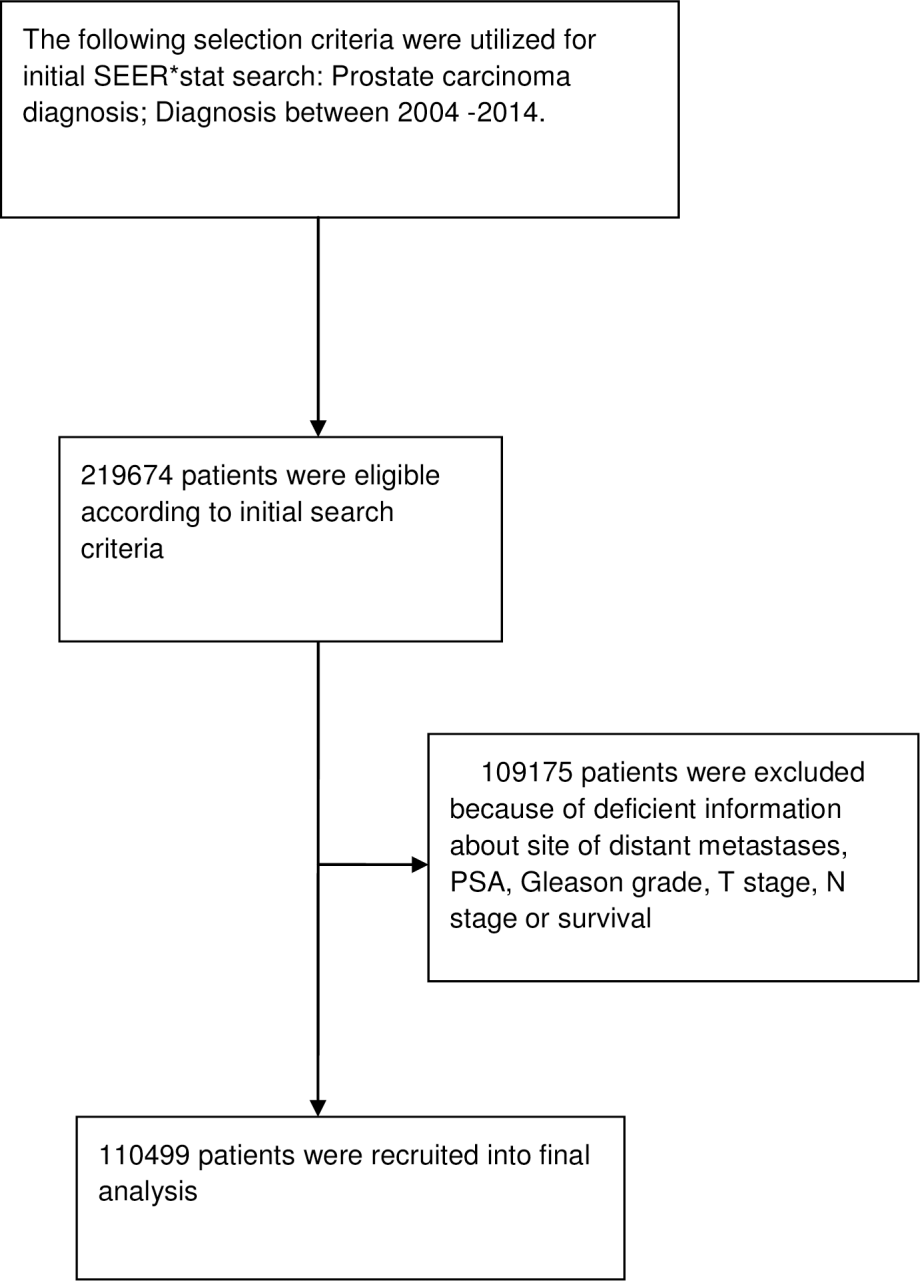
Flowchart showing the selection process of eligible patients into the current study.

“Fig 1” details the selection process of eligible patients into this study. A total of 110499 eligible patients with prostate cancer were finally identified in the period from 2004–2014 and were included into the analysis. All patients have complete information about TNM 6^th^ edition, PSA level as well as Gleason grade/grade group. Adenocarcinoma, NOS (not otherwise specified) represents the majority of cases (99.5%), other variants represent 0.5%.Age group < 70 years was 59.5%; while age group ≥70 years was 40.5%.Table 2 summarizes all clinicopathological characteristics of the included cohort. Distribution of the patients according to AJCC 6^th^, 7^th^ and 8^th^ editions was also reported. Moreover, classification of patients according to M stage were also reported (M0, M1a (non regional lymph nodes), M1b (bone metastases), M1c (other sites of metastases).Neither radiotherapy nor systemic therapy was reliably reported in the SEER database to be included in the analysis. All included patients did not undergo any form of radical surgery to the prostate. Median follow-up was 26 months(ranges from 1–131 months).

**Table 2:** Baseline characteristics of included patients in the study (110449 patients)**.

Parameter	Number (%)
Age	
<70 years	65794 (59.5%)
≥ 70 years	44655 (40.5%)
Race	
White	81878 (74.1%)
Black	20078 (18.2%)
Others	5701 (5.2%)
Unknown	2792 (2.5%)
Grade group	51231 (46.4%)
1	24775 (22.4%)
2	12058 (10.9%)
3	11556 (10.5%)
45	10829 (9.8%)
PSA	
<10	78572 (71.1%)
≥10- <20	17520 (15.9%)
≥20	14357 (13%)
Histological subtypes	
Adenocarcinoma, NOS	109875 (99.5%)
Other variants	574 (0.5%)
AJCC stage groups 6^th^ edition*	
I	346 (0.3%)
II	100774 (91.2%)
III	2452 (2.2%)
IV	6877 (6.2%)
AJCC stage groups 7^th^ edition*	
I	42528 (38.5%)
IIA	36986 (33.5%)
IIB	21606 (19.6%)
III	2504 (2.3%)
IV	6825 (6.1%)
AJCC stage groups 8^th^ edition*	
I	42528 (38.5%)
IIA	6817(6.2%)
IIB	22270 (20.2%)
IIC	16895 (15.3%)
IIIA	6605 (6%)
IIIB	1739 (1.6%)
IIIC	7165 (6.5%)
IVA	1197 (1.1%)
IVB	5233 (4.7%)
M stage	
M0	105216 (95.3%)
M1a (non regional lymph nodes)	362 (0.3%)
M1b (bone)	4051 (3.7%)
M1c (other sites)	820 (0.7%)

*AJCC: American Joint Committee on Cancer

**PSA laboratory examination unit was ng/ml.

### b. Survival outcomes

**Fig 2:**
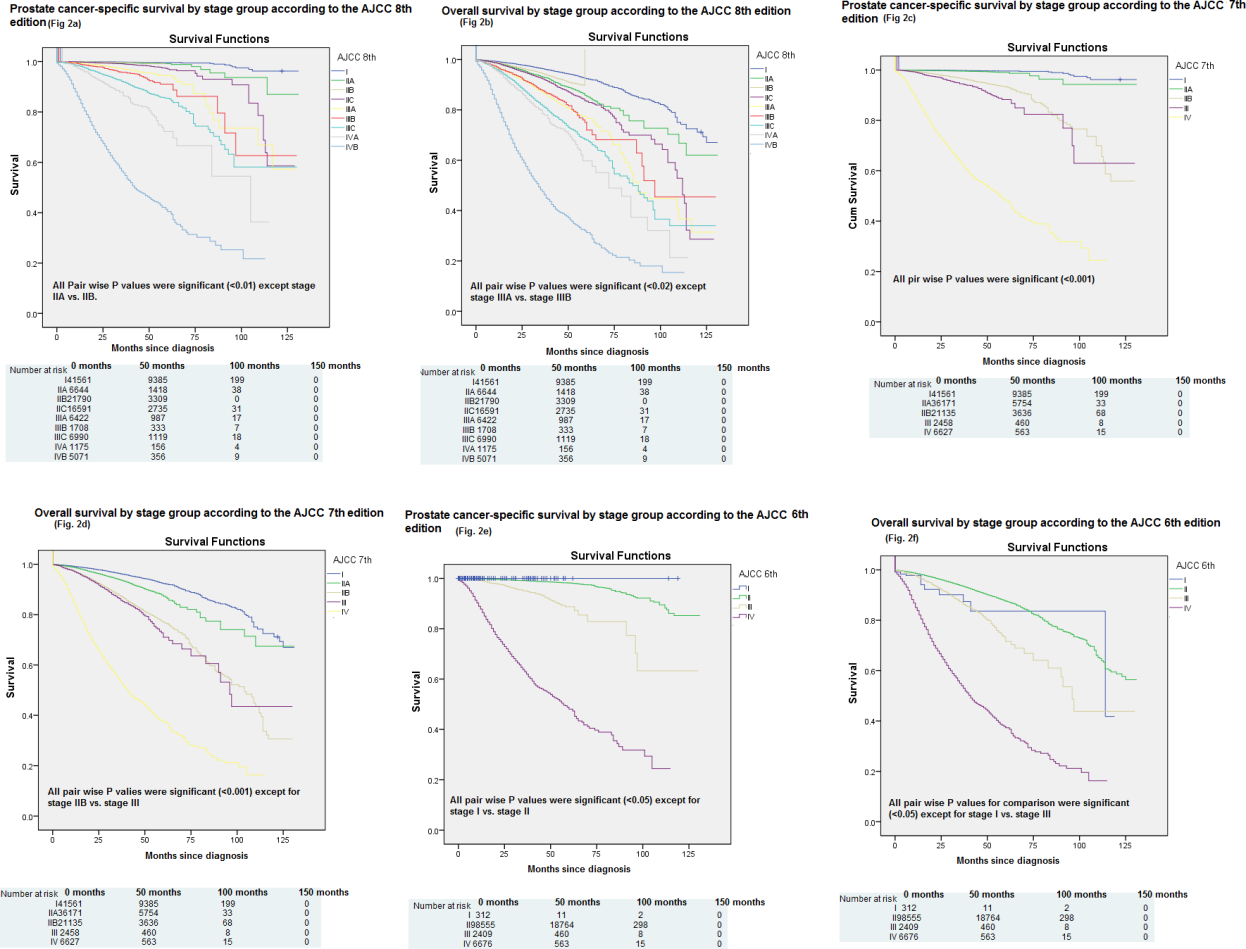
Kaplan–Meier curve of: a) cancer-specific survival according to the 8^th^ edition; b) overall survival according to the 8^th^ edition; c) cancer-specific survival according to the 7^th^ edition; d) overall survival according to the 7^th^ edition; e) cancer-specific survival according to the 6^th^ edition; f) overall survival according to the 6^th^ edition.

Cancer-specific and overall survivals were compared according to AJCC 6^th^, 7^th^ and 8^th^ staging systems. Pair wise comparison between all different stages with log rank testing was conducted. For cancer- specific survival according to 8^th^ AJCC, all pair wise P values for comparison were significant (<0.01) except for stage IIA vs. IIB; while for overall survival according to 8^th^ AJCC, all pair wise P values for comparison were significant (<0.02) except for stage IIIA vs. IIIB “Fig 2A and 2B”. For cancer-specific survival according to 7^th^ AJCC, all pair wise P values for comparison were significant (<0.001); while for overall survival according to 7^th^ AJCC, all pair wise P values for comparison were significant (<0.01) except for stage IIB vs. III “Fig 2C and 2D”. For cancer-specific survival according to 6^th^ AJCC, all pair wise P values for comparison were significant (<0.05) except for stage I vs. stage II; while for overall survival according to 6^th^ AJCC, all pair wise P values for comparison were significant (<0.05) except for stage I vs. III “Fig 2C and 2D” (notably in overall survival assessment according to 6^th^ AJCC, stage I has worse overall survival compared to stage II).

Results of c-index assessment for cancer-specific survival for the three editions were as follows: c-index for AJCC 6^th^ edition was 0.816; c-index for AJCC 7^th^ edition was 0.897; c-index for AJCC 8^th^ edition was 0.907. On the other hand, results of c-index assessment for overall survival for the three editions were as follows: c-index for AJCC 6^th^ edition was 0.627; c-index for AJCC 7^th^ edition was 0.704; c-index for AJCC 8^th^ edition was 0.710.

### c. Proposed modifications to the AJCC 8^th^ edition

**Fig 3:**
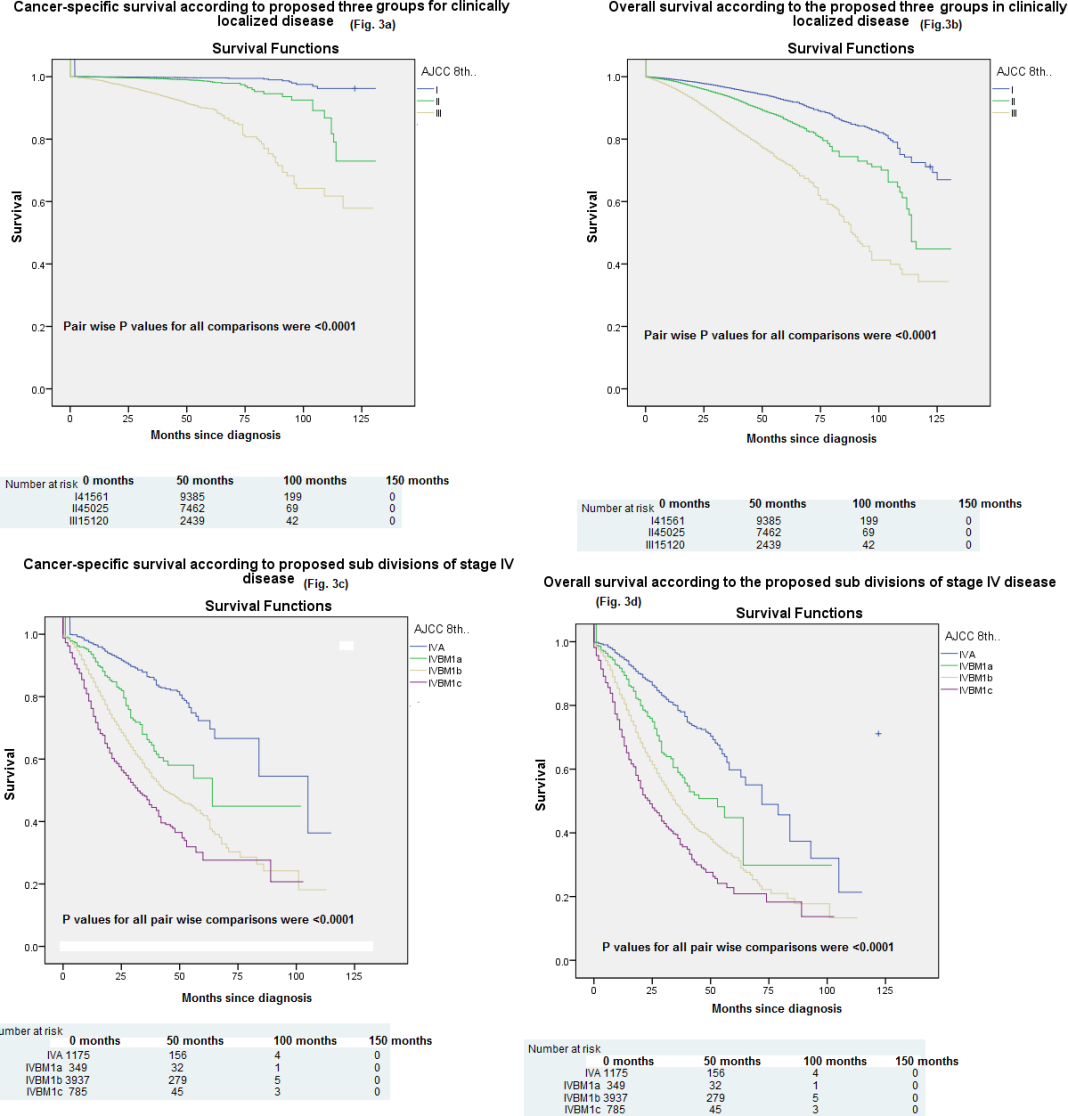
Kaplan–Meier curve of: a) cancer-specific survival according to the three risk groups for clinically localized disease; b) overall survival according to the three risk groups for clinically localized disease; c) cancer-specific survival according to the proposed sub-divisions of stage IV disease; d) overall survival according to the proposed sub-divisions of stage IV disease.

Based on the results above, two sets of modifications to the AJCC 8^th^ edition were formulated and proposed. For clinically localized disease (i.e. T any, N0, M0), three risk groups were proposed corresponding to stage I, stage II (IIA/IIB/IIC) and stage III (IIIA/IIIB/IIIC). Pair wise comparison between all different stages with log rank testing was conducted for both cancer-specific and overall survival. For both cancer-specific and overall survival, all pair wise P values for comparisons were <0.0001 “Fig 3A and 3B”.

Moreover, all P values for pair wise comparisons in a multivariate model of factors affecting cancer-specific and overall survival (adjusted for age, race, marital status and year of diagnosis) were also significant (P<0.0001) (Table 3).

**Table 3:** A multivariate Cox proportional model adjusted for age, race, year of diagnosis and marital status showing pair wise comparisons between the three stages of clinically localized disease (stage I,II,III)(AJCC 8^th^).

Comparison	Cancer-specific survival		Overall survival	
	HR (95% CI)	P value	HR (95% CI)	P value
Stage II vs. stage I	2.945 (2.313–3.750)	<0.0001	1.582 (1.485–1.684)	<0.0001
Stage I vs. stage III	0.044 (0.035–0.055)	<0.0001	0.293 (0.274–0.314)	<0.0001
Stage II vs. stage III	0.130 (0.113–0.150)	<0.0001	0.464 (0.438–0.492)	<0.0001

For advanced prostate cancer (N1 orM1 disease), further sub-staging was proposed according to M1 sub-stage (i.e.N1, M1a, M1b and M1c). Pair wise comparison between these new sub-stages with log rank testing was conducted for both cancer-specific and overall survival. For both cancer-specific and overall survival, all pair wise P values for comparisons were <0.0001 “Fig 3C and 3D”. Moreover, all P values for pair wise comparisons in a multivariate model of factors affecting cancer-specific and overall survival (adjusted for age, race, grade group, baseline PSA level, marital status and year of diagnosis) were also significant (P<0.0001) (Table 4). Additionally, Table 5 summarized five year cancer-specific survival rates according to the proposed sub-stages for advanced disease.

**Table 4:** A multivariate Cox proportional model adjusted for age, race, and year of diagnosis, marital status, grade group and baseline PSA showing pair wise comparisons between sub-stages of stage IV disease (AJCC 8^th^).

Comparison	Cancer-specific survival		Overall survival	
	HR (95% CI)	P value	HR (95% CI)	P value
Stage IVA vs. stage IVBM1a	0.491 (0.369–0.653)	<0.0001	0.606 (0.478–0.769)	<0.0001
Stage IVBM1a vs. stage IVBM1b	0.603 (0.479–0.760)	<0.0001	0.633 (0.518–0.714)	<0.0001
Stage IVBM1b vs. stage IVBM1c	0.682 (0.603–0.772)	<0.0001	0.690 (0.619–0.769)	<0.0001

**Table 5:** Five year cancer-specific survival rates among patients with stage IV disease (AJCC 8^th^).

Stage	Five year CSS rate*	SE*
IVA	72%	0.03
IVB(M1a)	54%	0.05
IVB(M1b)	43%	0.02
IVB(M1c)	30%	0.03

*CSS: cancer-specific survival; SE: standard error.

## 5. Discussion

The current study provided an external validation to the prognostic performance of the AJCC 8^th^ staging system for prostate cancer. Compared to older staging systems (6^th^ and 7^th^), the 8^th^ system is more discriminatoryand the new sub-stages introduced within the 8^th^ system are prognostically relevant. Additionally, stages I, II and III in the 8^th^system correspond to three distinct risk groups in the clinically localized setting. Moreover, new sub-stages should be introduced based on the site and extent of metastatic disease in the advanced setting.

Numerous prognostic tools have been proposed for prostate cancer both in the clinically localized as well as advanced settings. These models were evaluated by the committee of the precision medicine core of the AJCC [12, 13]. However, none of the models evaluating clinically localized disease were endorsed by the AJCC because of a reported lack of validation of survival impact. The current SEER analysis provided a survival validation for a three-risk groups system in the localized setting based on the 8^th^ AJCC system. Moreover, only two of the models in advanced disease were endorsed by the AJCC [14, 15]. These models deal with castrate-resistant prostate cancer rather than treatment-naïve disease.

P values for pair wise comparisons between different stages were not consistently significant between cancer-specific and overall survival. This may be ascribed to unknown differences in baseline co-morbidities which may have contributed to an imbalance of non cancer-related deaths and hence inconsistent P value for both endpoints.

Major limitations of this analysis include: 1) No sufficiently reliable information about both systemic and radiation treatments in the SEER database. The absence of sufficient treatment details calls for extra caution when dealing with the results of this analysis particularly with regards to the proposals for combining stages IIA/IIB/IIC or stages IIIA/IIB/IIC. Some of the observed overlaps among these sub-stages may be related to unknown treatment differences rather than inherent biological similarity.2) No information about performance status and co-morbidities of the included patients; accordingly, the analysis has been based on cancer-specific in addition to overall survival in order to avoid the potential confounding effect resulting from unknown associated co-morbidities.

Following the publication of the 7^th^ edition of TNM staging system for prostate cancer, a number of validation studies have been published and they showed an evidence of improvement for the 7^th^ system compared to the 6^th^ system [16, 17].

The 8^th^ edition of the AJCC staging system places more emphasis on the histological grade as a determinant factor in the stage grouping. The more enhanced role of disease biology in the staging is based on numerous analyses which point out the pivotal role of disease biology in determining prostate cancer outcomes [18, 19].

Current study shows that c-index for AJCC 8^th^ staging system is better than that of AJCC 7^th^ and 6^th^ staging systems. This indicates the improvement of discriminatory performance of the 8^th^ staging system compared to previous systems; and indicates also that with proper integration of biological information, the prognostication of many solid tumor patients may improve.

Current analysis reveals the heterogeneity in prognosis of patients with stage IV disease. This is in line with previous population-based studies [20, 21]. Although the traditional TNM staging classifies M1 disease into three sub-stages (M1a: non regional lymph nodes; M1b: bone metastases; M1c: other sites), the AJCC stage grouping did not sub-stage metastatic patients based on these M1 sub-stages despite evidence of survival differences based on the site and pattern of distant metastases [22]. The current study provided additional evidence on the importance of the site of metastases on survival and called for sub-classification of stage IV patients according to the site/extent of metastases.

Given the evidence of impact of tumor grade and PSA level on the outcomes of advanced disease, further refinements in the sub-classification of advanced disease may also be achieved by incorporating grade group and PSA levels into the staging of advanced disease (just like it was incorporated into the staging of clinically localized disease) [5]. This is best exemplified by the recently published prostascore model which incorporated anatomical extent of the metastases, baseline PSA and grade group into a simplified prognostic model for treatment-naïve advanced prostate cancer [23].

In conclusion,compared to older staging systems (6^th^ and 7^th^), the 8^th^ system is more discriminatoryand the new sub-stages introduced within the 8^th^ system are prognostically relevant.

## References

[1] globocan.iarc.fr. Last accessed on 27/11/2016

[2] Attard G, Parker C, Eeles RA, Schroder F, Tomlins SA, Tannock I, et al Prostate cancer. Lancet. 2016;387(10013):70–82. Epub 2015/06/16. doi: 10.1016/S0140-6736(14)61947-4 . 26074382 10.1016/S0140-6736(14)61947-4

[3] Gillessen S, Omlin A, Attard G, de Bono JS, Efstathiou E, Fizazi K, et al Management of patients with advanced prostate cancer: recommendations of the St. Gallen Advanced Prostate Cancer Consensus Conference (APCCC) 2015. Ann Oncol. 2015:mdv257. 10.1093/annonc/mdv257 PMC4511225 26041764

[4] Amin MB, Greene FL, Edge SB, Compton CC, Gershenwald JE, Brookland RK, et al The Eighth Edition AJCC Cancer Staging Manual: Continuing to build a bridge from a population‐based to a more “personalized” approach to cancer staging. CA Cancer J Clin. 2017;67(2):93–9. doi: 10.3322/caac.21388 28094848 10.3322/caac.21388

[5] Rusthoven CG, Carlson JA, Waxweiler TV, Yeh N, Raben D, Flaig TW, et al., editors. The prognostic significance of Gleason scores in metastatic prostate cancer. Urologic Oncology: Seminars and Original Investigations; 2014: Elsevier. 10.1016/j.urolonc.2014.01.004 24629494

[6] Izumi K, Ikeda H, Maolake A, Machioka K, Nohara T, Narimoto K, et al The relationship between prostate-specific antigen and TNM classification or Gleason score in prostate cancer patients with low prostate-specific antigen levels. Prostate. 2015;75(10):1034–42. Epub 2015/03/11. doi: 10.1002/pros.22985 . 25753899 10.1002/pros.22985

[7] Abdel-Rahman O. Validation of American Joint Committee on Cancer eighth staging system among prostate cancer patients treated with radical prostatectomy. Ther Adv Urol. 0(0):1756287217737706 doi: 10.1177/1756287217737706 10.1177/1756287217737706 PMC5805006 29434671

[8] Pierorazio PM, Walsh PC, Partin AW, Epstein JI. Prognostic Gleason grade grouping: data based on the modified Gleason scoring system. BJU Int. 2013;111(5):753–60. Epub 2013/03/08. doi: 10.1111/j.1464-410X.2012.11611.x ; PubMed Central PMCID: PMCPmc3978145. 23464824 10.1111/j.1464-410X.2012.11611.x PMC3978145

[9] Schymura MJ, Sun L, Percy-Laurry A. Prostate cancer collaborative stage data items—their definitions, quality, usage, and clinical implications: a review of SEER data for 2004–2010. Cancer. 2014;120 Suppl 23:3758–70. Epub 2014/11/21. doi: 10.1002/cncr.29052 . 25412388 10.1002/cncr.29052

[10] Surveillance, Epidemiology, and End Results (SEER) Program (www.seer.cancer.gov) SEER*Stat Database: Incidence—SEER 18 Regs Research Data + Hurricane Katrina Impacted Louisiana Cases, Nov 2016 Sub (1973–2014 varying)—Linked To County Attributes—Total U.S., 1969–2015 Counties, National Cancer Institute, DCCPS, Surveillance Research Program, Surveillance Systems Branch, released April 2017, based on the November 2016 submission.

[11] Epstein JI, Egevad L, Amin MB, Delahunt B, Srigley JR, Humphrey PA, et al The 2014 International Society of Urological Pathology (ISUP) consensus conference on Gleason grading of prostatic carcinoma: definition of grading patterns and proposal for a new grading system. The American journal of surgical pathology. 2016;40(2):244–52. doi: 10.1097/PAS.0000000000000530 26492179 10.1097/PAS.0000000000000530

[12] Buyyounouski MK, Choyke PL, McKenney JK, Sartor O, Sandler HM, Amin MB, et al Prostate cancer–major changes in the American Joint Committee on Cancer eighth edition cancer staging manual. CA Cancer J Clin. 2017:n/a-n/a. doi: 10.3322/caac.21391 28222223 10.3322/caac.21391 PMC6375094

[13] Kattan MW, Hess KR, Amin MB, Lu Y, Moons KGM, Gershenwald JE, et al American Joint Committee on Cancer acceptance criteria for inclusion of risk models for individualized prognosis in the practice of precision medicine. CA Cancer J Clin. 2016;66(5):370–4. doi: 10.3322/caac.21339 26784705 10.3322/caac.21339 PMC4955656

[14] Halabi S, Lin C-Y, Small EJ, Armstrong AJ, Kaplan EB, Petrylak D, et al Prognostic model predicting metastatic castration-resistant prostate cancer survival in men treated with second-line chemotherapy. J Natl Cancer Inst. 2013:djt280. 10.1093/jnci/djt280 PMC3833929 24136890

[15] Halabi S, Lin C-Y, Kelly WK, Fizazi KS, Moul JW, Kaplan EB, et al Updated prognostic model for predicting overall survival in first-line chemotherapy for patients with metastatic castration-resistant prostate cancer. J Clin Oncol. 2014;32(7):671–7. doi: 10.1200/JCO.2013.52.3696 24449231 10.1200/JCO.2013.52.3696 PMC3927736

[16] Zaorsky NG, Li T, Devarajan K, Horwitz EM, Buyyounouski MK. Assessment of the American Joint Committee on Cancer staging (sixth and seventh editions) for clinically localized prostate cancer treated with external beam radiotherapy and comparison with the National Comprehensive Cancer Network risk-stratification method. Cancer. 2012;118(22):5535–43. Epub 2012/05/01. doi: 10.1002/cncr.27597 ; PubMed Central PMCID: PMCPmc3410044. 22544661 10.1002/cncr.27597 PMC3410044

[17] Wong M, Yip C, Li H, Tan T, Kanesvaran R, Chowbay B, et al Assessment of the American Joint Committee on Cancer 7th Edition Staging for Localised Prostate Cancer in Asia Treated with External Beam Radiotherapy. Ann Acad Med Singapore. 2014;43:484–91. 25434618

[18] Glass TR, Tangen CM, Crawford ED, Thompson I. Metastatic carcinoma of the prostate: identifying prognostic groups using recursive partitioning. The Journal of urology. 2003;169(1):164–9. doi: 10.1097/01.ju.0000042482.18153.30 12478127 10.1016/S0022-5347(05)64059-1

[19] Gravis G, Boher J-M, Fizazi K, Joly F, Priou F, Marino P, et al Prognostic factors for survival in noncastrate metastatic prostate cancer: validation of the glass model and development of a novel simplified prognostic model. Eur Urol. 2015;68(2):196–204. doi: 10.1016/j.eururo.2014.09.022 25277272 10.1016/j.eururo.2014.09.022

[20] Muralidhar V, Mahal BA, Nguyen PL. Conditional cancer-specific mortality in T4, N1, or M1 prostate cancer: implications for long-term prognosis. Radiation Oncology. 2015;10(1):155. 26220664 10.1186/s13014-015-0470-0 PMC4518568

[21] Shukla ME, Yu C, Reddy CA, Stephans KL, Klein EA, Abdel-Wahab M, et al Evaluation of the current prostate cancer staging system based on cancer-specific mortality in the surveillance, epidemiology, and end results database. Clin Genitourin Cancer. 2015;13(1):17–21. doi: 10.1016/j.clgc.2014.07.003 25571871 10.1016/j.clgc.2014.07.003

[22] Gandaglia G, Karakiewicz PI, Briganti A, Passoni NM, Schiffmann J, Trudeau V, et al Impact of the Site of Metastases on Survival in Patients with Metastatic Prostate Cancer. Eur Urol. 2015;68(2):325–34. Epub 2014/08/12. doi: 10.1016/j.eururo.2014.07.020 . 25108577 10.1016/j.eururo.2014.07.020

[23] Abdel-Rahman O. Prostascore: A Simplified Tool for Predicting Outcomes among Patients with Treatment-naive Advanced Prostate Cancer. Clin Oncol (R Coll Radiol). 2017 Epub 2017/09/05. doi: 10.1016/j.clon.2017.08.003 . 28867136 10.1016/j.clon.2017.08.003

